# Growth and Performance of Guar (*Cyamopsis tetragonoloba* (L.) Taub.) Genotypes under Various Irrigation Regimes with and without Biogenic Silica Amendment in Arid Southwest US

**DOI:** 10.3390/plants12132486

**Published:** 2023-06-29

**Authors:** Alonso Garcia, Kulbhushan Grover, Dawn VanLeeuwen, Blair Stringam, Brian Schutte

**Affiliations:** 1 Department of Plant and Environmental Sciences, New Mexico State University, Las Cruces, NM 88003, USA; 2 Department of Economics, Applied Statistics and International Business, New Mexico State University, Las Cruces, NM 88003, USA; 3 Department of Entomology, Plant Pathology and Weed Science, New Mexico State University, Las Cruces, NM 88003, USA

**Keywords:** guar, clusterbean, irrigation, water use efficiency, biogenic silica, arid agriculture, guar seed yield

## Abstract

Guar is a potential crop that can be grown as a forage or as a seed crop in arid to semi-arid regions due to its low water requirements and tolerance to heat. Optimizing irrigation water use is important for making alternative crops such as guar a sustainable option. Amendments such as biogenic silica, a sedimentary rock from a biogenic source such as fossils, may help plants tolerate water stress due to reduced irrigation. The objective of the current study was to evaluate seed yield and attribute components and agronomic and physiological parameters for four guar genotypes (Matador, Kinman, Lewis, and NMSU 15-G1) under five drip irrigation regimes (I_1_-normal irrigation, I_2_-no irrigation at 75% pod formation, I_3_-no irrigation at 50% and 75% pod formation, I_4_-terminate irrigation at flowering, and I_5_-terminate irrigation at flowering + biogenic silica amendment) at Las Cruces in southern New Mexico, USA, from 2016 to 2018. On average, the I_1_ irrigation regime produced the highest guar seed yield (2715 kg ha^−1^) followed by I_5_ (2469 kg ha^−1^) from 2016 to 2018. As compared to the I_1_ regime, the I_2_ and I_3_ regimes resulted in a 20.8% and 23.4% decline in guar seed yield, respectively, on average from 2016 to 2018. The results suggest that the addition of biogenic silica might help to improve guar seed yield under reduced irrigation conditions and can produce comparable yields with an average of 300 mm of irrigation during the growing season in the southern New Mexico region of the Southwest US.

## 1. Introduction

Water scarcity is becoming an important issue worldwide, including in the arid and semi-arid environments of the Southwest US. Agriculture in major parts of the Southwest US, including New Mexico, depends on the water extracted from the Ogallala aquifer (the primary aquifer in the south-central US) for irrigation. For instance, in New Mexico, a total of 3740 thousand acre-feet of fresh water are extracted from the Ogallala aquifer per year [1]. High-volume withdrawals from the Ogallala aquifer, combined with low rainfall, have led to decreasing groundwater levels in regions including New Mexico [2,3]. 

Poor, sandy soils with low organic matter in the region make it difficult for growers to sustain crop production. High salt buildup is another issue facing agriculture in arid and semi-arid environments, including the desert in the Southwest US. Including alternative crops that can be successfully grown in limited water and high salt environments is an important strategy to sustain agriculture in such regions, including the Southwest US.

Guar (*Cyamopsis tetragonoloba* L.), also referred to as clusterbean, is a drought tolerant crop that is typically grown on sandy loam soils under hot and dry conditions [4]. Guar can be grown for forage, for fresh beans, or for seeds that are used to produce guar gum. Guar gum, which is approximately 37% of the endosperm, acts as a stabilizing agent in various food processing, pharmaceutical, and industrial processes [5]. Due to the use of guar gum as a gelling agent in oil and natural gas exploration, demand for and interest in guar gum has increased worldwide [6]. The US is the largest consumer of guar gum in the world, with most of its demands met through imports from India [7].

Domestic production of guar can help improve economic revenue and reduce the effect of drought on farms in the Southwest US [8]. Recent studies have shown that guar gum can be produced at lower prices than US import prices and produce lower greenhouse gas emissions than existing crops in the Southwest US [8]. Salinity tolerance of guar genotypes is another advantage that can improve its adaptability in the Southwest US [9,10,11].

Due to its potential as an alternative crop in the Southwest US and a renewed interest in guar production, research on various aspects of guar production has recently gained impetus in the Southwest US [9,10,11,12,13,14,15,16,17]. A few recent studies have focused on the response of guar to irrigation level and shown the adaptability of guar under deficit irrigation conditions [18,19]. However, no studies have looked at deficit irrigation impacts on guar in the study region in combination with soil amendments such as biogenic silica in the region.

Optimizing irrigation water use is important for making alternative crops such as guar a sustainable option. Excessive irrigation is not always desirable and may have a negative impact on seed yields of indeterminate crops, including guar, due to excessive vegetative growth and delayed seed formation [20,21]. For instance, optimal seed yield production in guar requires 900 mm of rainfall [22]. An excess of irrigation water can cause guar to decrease maximum seed yield [23]. Moreover, the impact of water stress on crop performance can depend on the growth stage at which the stress occurred. Reducing the irrigation at a later growth stage after the crop has established a well may help reduce water use with less impact on guar seed yield [18]. As guar is planted in early summer when the soil temperature is high in southern New Mexico [12], it might be a good strategy to ensure sufficient water availability during the early stage needed for good germination, crop emergence, and hence a good crop establishment. Once the plants have achieved sufficient growth, they can potentially better tolerate the water stress. Moreover, there are chances to receive moisture through precipitation in mid-summer in the region, thereby reducing the need to apply irrigation [12].

Guar is a legume that can fix nitrogen from the atmosphere using a symbiotic relationship with various species of rhizobia [24]. Although there are some rhizobium species that are able to survive under drought stress [25], guar production under drought conditions may benefit from a different range of products and amendments, such as biogenic silica. These soil amendments may help growers reduce their water usage.

Biogenic silica is a sedimentary rock that comes from a biogenic source, such as fossils. Several studies have reported the beneficial impact of biogenic silica under biotic and abiotic stress factors that may affect the growth of plants [26,27,28]. Biogenic silica is not considered an essential nutrient for plant growth [28], except for the Equisetaceae, or horsetail family, due to its high content of silicon [29].

Silica helps plants to tolerate stresses by improving physiological processes [27]. Biogenic silica has also been shown to increase leaf water potential [26] by causing the epidermis to form a double layer. Similarly, the addition of silica can increase the osmotic adjustment under water stress [30]. Furthermore, biogenic silica addition can help enhance amounts of antioxidant defense enzymes in rice (*Oryza sativa* L.) and thereby improve drought tolerance [31].

The benefits of silicon can vary among different legume species under abiotic and biotic stresses [32]. For instance, some legume species, such as pigeonpea *Cajanus cajan*, common bean *P. vulgaris*, and soybean *Glycine max*, can accumulate relatively high concentrations of silicon in their foliar tissues [33]. Silicon supplementation has been reported to promote root nodulation and the biosynthesis of foliar amino acids in lucerne, *Medicago sativa* [34].

There is no information available in the literature about the role of silica on guar growth under limited water conditions. We hypothesize that the addition of biogenic silica as an amendment would help guar plants improve their growth and yield under reduced irrigation.

The objective of the current study was to evaluate selected guar genotypes for their growth, physiological parameters, seed yield, and yield attributes under reduced irrigation with and without the addition of biogenic silica amendment in the southern New Mexico region of the Southwest US.

## 2. Results

### 2.1. Growth and Biomass

In general, the growth and biomass of guar plants were affected by irrigation regimes, with slightly varying results in different years. At maturity, in 2018, plant height under I_1_ was higher than plant height under any other irrigation regime. Additionally, I_1_ had a higher plant height than I_2_ in 2016 and higher than I_4_ in 2017 (Table 1). In 2016, I_5_ had a higher plant height than I_4_, I_3_, or I_2_. Among genotypes, Lewis and NMSU-15-G1 had significantly higher plant heights compared to Kinman in 2016 (Table 1).

Above ground biomass measurements at maturity also varied among the irrigation regimes. Among irrigation regimes, the numerically highest above ground dry biomass at maturity was recorded under I_1_ (2017 and 2018) and I_5_ (2016) (Table 1), although I_1_ and I_5_ did not differ significantly in any year. I_5_ had higher above-ground dry biomass than I_4_ in 2016 and 2017. Among genotypes, in 2016, above-ground dry biomass was higher for NMSU-15-G1 compared to Kinman and Matador (Table 1).

### 2.2. Physiological Parameters

Varying results were observed in the physiological parameters among different irrigation regimes when measured at different stages of plant growth during the study. For instance, when measured at 50% pod formation, the SPAD chlorophyll values did not differ among irrigation regimes in 2016 (Table 1). In 2017, SPAD chlorophyll values at 50% pod formation were higher for I_1_ compared to I_3_ and I_4_ regimes (Table 1). In 2018, I_5_ had the highest SPAD value at the 50% pod formation, while I_2_ had a lower SPAD than I_3_, I_4_, or I_5_. The I_5_ regime resulted in numerically higher SPAD chlorophyll values than I_4_ at 50% pod formation from 2016 to 2018, with the difference significant only in 2018 (Table 1).

Among genotypes, during 50% pod formation in 2016, the highest SPAD value was recorded under Matador, which was significantly higher than Kinman and NMSU-15-G1 (Table 1). In 2018, Lewis had significantly higher SPAD values compared to Matador and NMSU-15-G1 (Table 1).

At 100% pod formation, among irrigation regimes, I_5_ had higher (2016 and 2017) or similar (2018) SPAD values as compared to I_1_ (Table 1) and had higher SPAD than I_4_ in all three years.

Physiological parameters, including photosynthetic rate, stomatal conductance, and transpiration rate, also showed varying results among the irrigation regimes. For instance, at the 75% pod formation stage in 2018, I_1_ and I_2_ had a higher photosynthetic rate than I_3_ and I_4_, with I_5_ having an intermediate value that did not differ significantly from any other irrigation regime (Table 2). Among genotypes, the lowest photosynthetic rate was observed for Kinman compared to other genotypes in 2016 (Table 2).

Results for stomatal conductance indicated significant differences among irrigation regimes only in 2018, with the I_1_ regime resulting in higher stomatal conductance compared to other irrigation regimes except for I_2_ (Table 2). In 2018, at the 75% pod formation stage, the transpiration rate was higher under the I_1_ regime than the I_3_ or I_4_ regimes (Table 2). Among genotypes, Kinman was lower compared to other genotypes only for 2016 (Table 2).

In the current study, biogenic silica seemed to have a positive effect on guar physiological parameters. In 2018, SPAD under I_5_ was higher than under I_4_ at the 50% pod formation stage in 2018 and in all three years at the 100% pod formation stage. At 75% pod formation in 2018, I_4_ had a lower photosynthetic rate than I_1_, while I_1_ and I_5_ did not differ significantly.

### 2.3. Water Use Efficiency

Intrinsic water use efficiency (WUE_i_) did not differ among irrigation regimes and genotypes from 2016 to 2018 (Table 3). Instantaneous water use efficiency (WUE_inst_) was greater under the I_5_ regime in 2017 compared to the other irrigation regimes except for I_4_ (Table 3). Results could possibly be attributed to the capacity of guar to withstand drought.

Agronomic water use efficiency was significantly higher under I_5_ compared to all other irrigation regimes in 2016 (Table 3). The I_5_ regime also had a significantly higher WUE_y_ compared to the I_4_ regime in 2018 (Table 3). In 2016, the genotype NMSU-15-G1 had significantly lower WUE_y_ compared to other genotypes at maturity (Table 3).

### 2.4. Yield Attributing Characteristics and Seed Yield

While significance patterns varied for different yield attributing characteristics and years when recorded under different irrigation regimes and genotypes during the study, I_1_ and I_5_ had numerically higher values than other regimes for clusters per plant, pods per plant, seeds per plant, and seed yield in all years. For instance, a higher number of clusters per plant was observed under I_5_ as compared to other irrigation regimes in 2016, while no significant differences were observed among irrigation regimes in 2017 and 2018 (Table 4). Among genotypes, Kinman and NMSU-15-G1 had a significantly higher number of clusters per plant as compared to Matador in 2016, while clusters per plant did not differ among genotypes in 2017 and 2018 (Table 4).

In 2016, the number of pods per plant under I_5_ was higher than under I_2_, I_3_, or I_4_, while in 2018, I_1_ had a higher number of pods per plant than I_2_, I_3_, or I_4_ (Table 4). In 2017, I_3_ had a lower number of pods per plant than I_1_ and I_5_ (Table 4).

The number of seeds per pod did not differ among irrigation regimes from 2016 to 2018 (Table 4). Among genotypes, Kinman produced the lowest number of seeds per pod, while Matador produced the highest number of seeds per pod from 2016 to 2018 (Table 4).

The total number of seeds per plant was higher under I_5_ compared to I_4_ and I_3_ irrigation regimes in 2016 and 2017, while differences were not significant in 2018 (Table 4). The total number of seeds per plant did not differ among genotypes from 2016 to 2018 (Table 4).

The 1000-seed weight did not differ among irrigation regimes in 2016 and 2018 (Table 5). In 2017, I_5_ had a higher 1000-seed weight than I_2_, I_3_, or I_4_ (Table 5). Among genotypes, NMSU-15-G1 had a significantly higher 1000-seed weight compared to other genotypes in 2016 and 2017 (Table 5). The 1000-seed weight under Matador was significantly lower compared to other genotypes in 2018 (Table 5).

The harvest index (HI) was lower under I_5_ compared to other irrigation regimes in 2016 (Table 5). In 2017, however, the I_5_ and I_4_ regimes recorded higher HI compared to I_1_ and I_2_ (Table 5). Among genotypes, the highest HI was recorded under Kinman compared to Lewis and NMSU-15-G1 in 2017 and 2018 (Table 5).

Guar seed yields varied among the irrigation regimes (Table 5). In general, higher levels of guar seed yield were obtained under I_1_ (Table 5) as compared to I_2_, I_3_, and I_4_). Results were mixed for the I_1_–I_5_ comparison, with I_5_ having a numerically higher but not significantly different seed yield than I_1_ in 2016 but a lower seed yield than I_1_ in 2018. When compared to I_4_, the addition of biogenic silica in I_5_ resulted in a significant increase in seed yield in 2016 (Table 4). Similar trends were observed in 2017 and 2018, with numerically (but not significantly) higher seed yields under I_5_ than I_4_ (Table 5). Among genotypes, no significant differences were observed in their seed yield in 2017 and 2018 in Las Cruces. In 2016, NMSU-15-G1 resulted in a significantly lower seed yield as compared to other genotypes.

## 3. Materials and Methods

### 3.1. Experimental Site and Design Description

The study was conducted at the Fabian Garcia Science Center (32°16′ N, 106°46′ W and an elevation of 1186 m) of New Mexico State University, Las Cruces, in southern NM, from 2016 to 2018. An annual precipitation of 185 mm fell from 2016 to 2018 in Las Cruces. Mean annual temperatures in New Mexico range from 18 °C to 4.5 °C [23]. The research plot had a sandy loam soil texture. The field was irrigated using a sub-surface drip tape (T-tape, Rivulis irrigation in 2016 and 2017, and T-tape, POWERTAPE in 2018). The amount of irrigation water applied was calculated by multiplying the total number of hours irrigated by the drip emitter flowrate.

The experiment was laid out in a split-plot design with five irrigation regimes as main plot factors and four guar genotypes as sub-plot factors. The four genotypes were randomly assigned to four sub-plots contained within each irrigation regime plot within each block, for a total of four replications (blocks). The four genotypes included three released varieties (Matador, Kinman, and Lewis) and one test line (NMSU-15-G1) that were selected based on best yield performance in previous studies at the same location [12].

The irrigation regimes included: (1) I_1_-normal irrigation (regular schedule with no skipped irrigation at any stage), (2) I_2_-no irrigation at 75% pod formation (skipped irrigation at 75% pod formation), (3) I_3_-no irrigation at 50% and 75% pod formation (skipped irrigations at 50% and 75% pod formation stages), (4) I_4_-terminate irrigation at flowering (no irrigation was applied after flowering stage), and (5) I_5_-terminate irrigation at flowering + biogenic silica (no irrigation was applied after flowering stage; biogenic silica amendment was applied).

The I_5_ regime included a total of three applications of biogenic silica (SiO_2_). The first application of biogenic silica was side-dressed at the trifoliate stage at a rate of 2500 ppm using a water pot. The second and third applications were applied as foliar sprays with a backpack sprayer at 50% pod formation and 75% pod formation using a rate of 2800 ppm.

### 3.2. Field Preparation

Raised beds of 1.02 m width were prepared and irrigated prior to planting. Guar seeds were manually planted at a depth of 2.5 cm in two rows on the raised beds. Hand thinning was conducted one week after emergence to maintain a plant population of 264,000 plants/ha. Weeds were removed manually using hand hoes. An insecticide was used to control leaf miners in 2016: MustangMax 279-3327 (FMC Agricultural Products, Philadelphia, PA, USA) at a rate of 25 g of active ingredient per ha in June, followed by a spray of Radiant 62719-545 (Dow AgroSciences, Indianapolis, IN, USA) in July at a rate of 40 g of active ingredient per ha.

### 3.3. Data Collection

Physiological measurements, including photosynthetic rate, stomatal conductance, and plant transpiration rate, were recorded on the youngest mature leaf during the 75% pod formation stage using a portable photosynthesis system (LI-COR 6400, Lincoln, NE, USA, 68504). The LI-COR 6400 system was calibrated, and the light source used was 6400-02b. Quantum flux photosynthetic active radiation (PAR) used was 1000 µmol photons m^−2^ s^−1^ and a CO_2_ concentration of 400 ± 10 µmol mol^−1^. All of these observations were taken on a clear, sunny day between 10:00 am and 1:30 pm.

Water use efficiencies (WUE) were calculated from data derived from the portable photosynthetic system. Such data included CO_2_ assimilation (A_N_), transpiration rate (E), and stomatal conductance (gs). Instantaneous water use efficiency was calculated as the ratio between A_N_ and E (A_N_/E), while intrinsic water use efficiency was calculated as the ratio between A_N_ and gs (A_N_/gs) [35]. Intrinsic water use efficiency (WUE_i_) refers to the photosynthetic water use efficiency or carbon assimilation in comparison to the stomatal conductance [36]. Instantaneous water use efficiency (WUE_inst_) is related to the comparison between the rate of photosynthesis and the rate of transpiration [37]. Agronomic water use efficiency (WUE) is defined as the amount of water used per season for the total seed yield during the season for the guar crop [38].

A Field Scout SPAD 502Plus chlorophyll meter (Spectrum Technologies, Inc., Aurora, IL, USA, 60504) was used to determine chlorophyll content in the leaves at 50% and 100% pod formation stages in 2016, 2017, and 2018.

Plant height, above-ground dry biomass, clusters per plant, seeds per pod, pods per plant, seeds per plant, 1000 seed weight (g), harvest index, and seed yield (kg ha^−1^) were recorded at maturity stage. Plant height was measured from the soil surface to the top of the plant using a meter scale. Above ground dry biomass weight was recorded after drying the samples at 55 °C for 72 h. Clusters per plant, seeds per pod, and pods per plant were counted on two randomly collected plants from each sub-plot. The total number of seeds per plant was calculated by multiplying seeds per pod by pods per plant. In 2016, for a 1000-seed weight, seeds were counted using a seed counter (Seedburo Equipment Co., Des Plaines, IL, USA, 60018). In 2017 and 2018, the seed counter used was the SLY-A automatic seed counter (Zhejiang Top Instrument Co., Ltd., Hangzhou, China, 311000).

For aboveground dry biomass measurements, guar plants were harvested from one-m^2^ sections in each plot and then oven dried at 55 °C for 72 h, and dry weight was recorded. Seed weight was also recorded from these samples, and a harvest index was calculated by dividing the seed weight per m^2^ by the aboveground dry biomass per m^2^. Pods were manually threshed to collect the seeds using a screen to avoid any seed losses. Finally, the threshed samples were cleaned using a clipper office tester (Clipper Separation Technologies, Bluffton, IN, USA, 46714).

The total seed weight from each sub-plot was also collected by manually harvesting the whole plants and collecting all the pods. The collected pods were then threshed using a Large Vogel plot thresher (LVPT, ALMACO Inc., Nevada, IA, USA, 50201).

### 3.4. Statistical Analysis

Data from the four replications were analyzed using a mixed model analysis for a split-plot design with whole plot factor irrigation regime and subplot factor genotype. The mixed model included fixed effects for irrigation regime, genotype, and their interaction. The random effects included block and the whole plot factor experimental unit (i.e., the block*irrigation regime interaction). Data were analyzed for each individual year using SAS PROC MIXED software version 9.4 (SAS Institute Inc., Cary, NC, USA, 2016). When the F-test was found to be significant (*p* = 0.05), a pairwise means separation test was conducted using Fisher’s protected LSD with PDMIX MACRO [39].

## 4. Discussion

Water scarcity in semi-arid environments, including the Southwest US, needs to be addressed. Crops such as guar that can grow under limited water conditions can help sustain agriculture in such regions. The current study focused on investigating the response of selected guar genotypes to various irrigation regimes resulting in water stress at different stages during the reproductive phase with and without an amendment of biogenic silica.

Most of the growth and yield parameters of guar were affected by irrigation regimes, with slightly varying results in different years. In general, growth parameters such as plant height and above-ground dry biomass at maturity were higher under the normal irrigation (I_1_) regime, where no stress was experienced by guar plants, as compared to the reduced irrigation regimes (I_2_, I_3_, and I_4_) (Table 1). These results are in accordance with previous studies that reported similar results [19,40]. It is interesting to note that although the irrigation regimes differed in imposing water stress during the reproductive stage, the water stress impacted plant height and above-ground biomass. This is probably due to the indeterminate growth type of guar plants, which can grow vegetatively even after the reproductive stage has set in [14].

Application of biogenic silica in the regime with early termination of irrigation (I_5_) resulted in higher plant height and above-ground biomass as compared to the regime with early termination of irrigation that received no biogenic silica (I_4_), indicating a positive impact of biogenic silica on plant growth (Table 1). Similar results indicating the benefits of biogenic silica have been reported on other plants as well [26,27]

Physiological parameters such as SPAD chlorophyll, photosynthetic rate, stomatal conductance, and transpiration rate of guar showed varying results among different irrigation regimes when measured at different stages of plant growth during the study. In general, SPAD chlorophyll values were higher under the normal irrigation (I_1_) regime, where no stress was experienced by guar plants, as compared to the reduced irrigation regimes (I_2_, I_3_, and I_4_) (Table 1). The early termination irrigation regime receiving the application of biogenic silica (I_5_) resulted in higher SPAD values than when no biogenic silica was applied to the early termination irrigation regime (I_4_). Moreover, SPAD chlorophyll values under I_5_ were either higher or similar to the I_1_ regime, indicating beneficial effects of biogenic silica under water stress conditions. Previous studies suggested that the application of silicon to cucumbers under drought helped increase levels of chlorophyll [41]. Similarly, other studies have reported positive impacts of biogenic silica application on physiological parameters such as osmotic adjustment [30], interception of light and leaf water potential [26], and photosynthetic activities [42].

Intrinsic and instantaneous water use efficiency indicate the photosynthetic efficiency of water use in relation to stomatal conductance and transpiration rate, respectively. There were either no differences (intrinsic) or slight differences (instantaneous) in water use efficiency among varied irrigation regimes, indicating that guar plants were able to adjust stomatal conductance and transpiration rates under water stress and, therefore, were able to withstand water stress or drought (Table 2 and Table 3).

The agronomic water use efficiency (WUE_y_), which is a measure of seed yield produced per unit of water used, however, was higher under I_5_ compared to all other irrigation regimes in 2016 and as compared to I_4_ in 2018, indicating the positive impact of biogenic silica on guar growth under water stress (Table 3). Application of silicon was also reported to increase the amount of water uptake and antioxidant enzymes in wheat [43].

The seed yield-attributing characteristics contribute to the final seed yields and therefore can impact the overall productivity of a crop. In the current study, the seed yield attributing characteristics, including clusters per plant, pods per plant, seeds per pod, and seeds per plant, were recorded under different irrigation regimes. Although differences were not always found to be significant among irrigation regimes, I_1_ and I_5_ generally had higher numerical values of these attributing characteristics than other irrigation regimes (Table 4). Previous studies have also reported pods per plant to be an important seed yield-attributing characteristic of guar under water stress conditions [44]. The I_5_ regime had a higher number of clusters per plant, pods per plant, and total number of seeds per plant than the I_4_ irrigation regime, showing the positive impact of biogenic silica on these yield-attributing characteristics.

**Figure 1:**
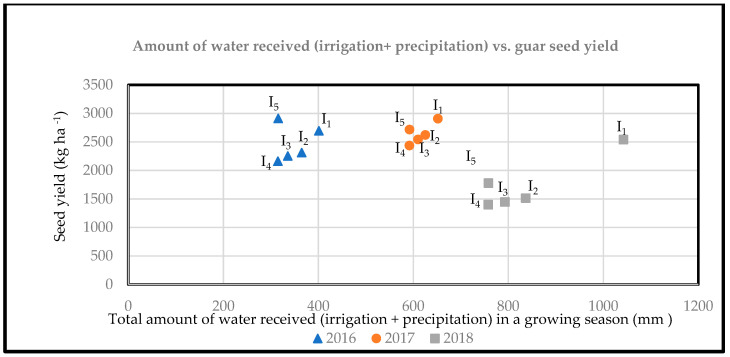
Amount of total water received (Rain + irrigation) vs. guar seed yield from 2016–2018 in Las Cruces, NM. I_1_-normal irrigation, I_2_-no irrigation at 75% pod formation, I_3_-no irrigation at 50% and 75% pod formation, I_4_-terminate irrigation at flowering, and I_5_-terminate irrigation at flowering + biogenic silica. The 50% and 75% pod formation stages at 57 and 72 days after planting, respectively.

The results showed that among irrigation regimes, in general, higher levels of guar seed yield were obtained under I_1_ (Table 5) as compared to I_2_, I_3_, and I_4_, which received an annual average of 552.4 mm of irrigation plus 146 mm of precipitation from 2016 to 2018 (Figure 1). Results were mixed for the I_1_–I_5_ comparison, with I_5_ having a numerically higher but not significantly different seed yield than I_1_ in 2016 but a lower seed yield than I_1_ in 2018. In southwestern New Mexico, the rain will most likely begin after the flowering stage if it is planted in mid-June for optimum guar development [12]. Earlier studies also showed that guar can achieve its optimum growth in non-irrigated areas with an annual rainfall ranging from 254 to 1016 mm [3], which is less or comparable to the total water needed per season for alfalfa (800–1600 mm), cotton (700–1300 mm), and potato (500–700 mm) [30].

While comparing the data from different years, guar seed yields obtained in 2018 were lower than in 2016 and 2017 (Figure 1). This was even though the total amount of water applied through irrigation was 160% greater in 2018 than in 2016. There was some rain received along with irrigation at emergence, but no rain or irrigation was received during the unifoliate in 2018. This relatively longer dry spell during the unifoliate stage could have affected the overall development of guar plants in 2018. Additionally, guar plants experienced a longer spell of dryness and heat, resulting in wilting symptoms at the 50% pod-formation stage, due to which extra irrigation at the 50% pod-formation stage was applied to all treatments in 2018. This was a key observation, indicating the importance of water availability at the pod-formation stage. This is in agreement with previous research indicating irrigation at mid-pod filling is critical for guar seed production [20].

The distribution of precipitation also seemed to play an important role in the growth and development of guar plants in the current study. For instance, although the total amount of rainfall received during the guar growing season was similar in 2016 and 2018, a lower guar seed yield was obtained even when the amount of irrigation was higher in 2018 than in 2016. This could potentially be due to the distribution of rainfall and the availability of sufficient amounts of water at regular intervals. A previous study conducted in Iran also showed that regular irrigation every three days was most efficient for guar seed production, indicating the importance of the distribution of available water through the growing season [45].

When averaged from 2016 to 2018, I_2_ resulted in a 20.8% decline in seed yield as compared to I_1_ (Figure 1). Similarly, I_3_ resulted in an average decline of 23.4% in guar seed yield in Las Cruces from 2016 to 2018. The lowest seed yield was obtained under I_4_ (Figure 1), which resulted in a 26.4% decline in seed yield as compared to I_1_ based on average yield.

It is interesting to note that I_5_ resulted in only a 9.1% decline in seed yield as compared to the highest yielding I_1_, when averaged from 2016–2018. When compared to I_4_, the addition of biogenic silica in I_5_ resulted in a significant increase in the seed yield and yield-attributing characteristics, including clusters per plant, pods per plant, and seeds per plant, in 2016 (Table 4). Similar trends were observed in 2017 and 2018, with numerically (but not significantly) higher seed yields under I_5_ than I_4_ (Figure 1) (Table 5). The positive impact of biogenic silica in improving the guar seed yield under I_5_ as compared to I_4_ could be attributed to its role in improving processes including metabolism and altering physiological activities such as osmotic adjustment [30], interception of light and leaf water potential [26], photosynthetic activities [42], and mineral uptake [46].

For the years in this study, it appears that if irrigation is terminated after flowering, adding biogenic silica may completely mitigate the effects of the reduced irrigation in some years but not in others. The results obtained from the current research using drip irrigation showed that guar can grow well in the region with an average of 300 mm of irrigation during the growing season.

## 5. Conclusions

This study recorded that reasonable guar seed production can be obtained under reduced irrigation conditions after flowering in southern New Mexico. On average, the I_1_ irrigation regime produced the highest guar seed yield (2715 kg ha^−1^), followed by I_5_ (2469 kg ha^−1^) from 2016 to 2018. As compared to the I_1_ regime, the I_2_ and I_3_ regimes resulted in a 20.8% and 23.4% decline in guar seed yield, respectively, on average from 2016 to 2018. Among genotypes, Kinman and Matador seemed to perform slightly better with higher growth and yield parameters than other genotypes; the seed yield differences, however, were not significant.

The results of this study suggest that the addition of biogenic silica might help to improve seed yield production under reduced irrigation conditions, such as early termination at flowering, in arid irrigated agriculture in southern New Mexico. The addition of biogenic silica seemed to help alleviate water stress and have a positive impact on guar seed yield. Under water stress, biogenic silica appeared to improve the chlorophyll content and yield attributing characteristics.

## Figures and Tables

**Table 1:** SPAD leaf chlorophyll content at 50% and 100% pod formation stages and plant height and above-ground dry biomass (AGDB) at maturity stages of four guar genotypes under five irrigation regimes from 2016 to 2018, Las Cruces, NM.

	SPAD Chlorophyll Content						Plant Height (cm)			AGDB (g/Plant)		
	50% Pod Formation Stage			100% Pod Formation Stage			Maturity			Maturity		
	2016	2017	2018	2016	2017	2018	2016	2017	2018	2016	2017	2018
Irrigation regime (I)												
I_1_	67.6	60.6 a	66.1 bc	68.8 b	53.6 b	63.6 a	90.4 ab	116.7 ab	87.2 a	157.6 ab	153.9 a	71.0 a
I_2_	69.7	59.3 ab	65.5 c	65.5 b	55.8 b	59.3 ab	78.2 c	125.3 a	67.2 b	110.7 b	132.2 ab	43.4 b
I_3_	68.4	55.7 c	67.8 b	68.4 b	54.7 b	56.6 b	80.7 bc	113.7 bc	65.0 b	127.0 b	107.0 b	36.0 b
I_4_	67.1	56.1 bc	67.9 b	69.5 b	52.5 b	57.3 b	82.3 bc	106.2 c	62.7 b	143.2 b	112.1 b	32.4 b
I_5_	67.3	57.8 abc	70.4 a	78.9 a	62.0 a	62.5 a	97.6 a	108.9 bc	69.3 b	198.4 a	150.1 a	51.7 ab
Standard Error	1.1	1.4	0.9	3.9	1.5	2.2	4.2	3.9	4.8	16.2	10.1	10.0
Genotype (G)												
Kinman	64.3 c	59.4	67.8 ab	68.9	56.3	62.1	81.2 b	112.0	71.0	127.6 b	122.2	48.4
Lewis	69.0 ab	56.9	69.6 a	72.3	54.4	61.2	89.1 a	117.6	72.1	153.1 ab	141.3	48.1
Matador	71.0 a	58.0	66.8 b	69.8	55.0	59.2	85.5 ab	113.0	69.0	135.9 b	133.0	45.5
NMSU-15-G1	67.9 b	57.1	66.0 b	69.8	57.0	57.0	87.6 a	114.0	69.0	173.0 a	127.7	45.7
Standard Error	1.0	1.4	0.9	3.7	1.2	2.1	3.3	3.2	2.5	12.7	10.7	8.8
Interaction (I × G)	NS	NS	NS	NS	NS	NS	NS	NS	NS	NS	NS	NS

Means within a column and particular effects not including letters or sharing a letter do not differ at α < 0.05 (Fisher’s F-protected LSD). I_1_-normal irrigation, I_2_-no irrigation at 75% pod formation, I_3_-no irrigation at 50% and 75% pod formation, I_4_-terminate irrigation at flowering, and I_5_-terminate irrigation at flowering + biogenic silica. The 50%, 75%, and 100% pod formation stages at 57, 72, and 100 days after planting, respectively; maturity stage at 120 days after planting. NS = Not significant at α < 0.05.

**Table 2:** Photosynthetic rate, stomatal conductance, and transpiration rate at 75% pod formation stage of four guar genotypes under five irrigation regimes from 2016 to 2018, Las Cruces, NM.

	Photosynthetic Rate (µmol m^−2^ s^−1^)			Stomatal Conductance (mol m^−2^ s^−1^)			Transpiration Rate (mmol m^−2^ s^−1^)		
	2016	2017	2018	2016	2017	2018	2016	2017	2018
Irrigation regime (I)									
I_1_	25.6	26.9	25.7 a	0.60	0.76	0.54 a	6.2	9.4 ab	9.4 a
I_2_	25.7	27.9	23.1 a	0.57	0.88	0.42 ab	6.0	10.2 a	9.0 ab
I_3_	26.1	26.2	17.1 b	0.59	0.82	0.21 c	6.0	9.5 ab	5.9 c
I_4_	24.9	26.3	18.2 b	0.49	0.69	0.25 bc	5.8	8.9 b	6.6 bc
I_5_	26.0	26.6	21.8 ab	0.66	0.61	0.33 bc	6.3	8.6 b	8.5 ab
Standard Error	0.7	0.9	1.8	0.07	0.09	0.07	0.6	0.5	0.8
Genotype (G)									
Kinman	23.8 b	26.0	20.7	0.48	0.74	0.33	5.6 b	9.8	7.8
Lewis	26.6 a	27.4	22.4	0.62	0.81	0.39	6.1 a	9.0	8.3
Matador	26.4 a	26.2	19.7	0.60	0.70	0.31	6.2 a	9.1	7.4
NMSU-15-G1	25.9 a	27.5	21.9	0.62	0.75	0.38	6.4 a	9.4	8.0
Standard Error	0.7	0.8	1.5	0.06	0.08	0.06	0.6	0.6	0.8
Interaction (I × G)	NS	NS	NS	NS	NS	NS	NS	NS	NS

Means within a column and particular effects not including letters or sharing a letter do not differ at α < 0.05 (Fisher’s F-protected LSD). I_1_-normal irrigation, I_2_-no irrigation at 75% pod formation, I_3_-no irrigation at 50% and 75% pod formation, I_4_-terminate irrigation at flowering, and I_5_-terminate irrigation at flowering + biogenic silica. The 50% and 75% pod formation stages at 57 and 72 days after planting, respectively. NS = Not significant at α < 0.05.

**Table 3:** Intrinsic water use efficiency (WUE_i_) and Instantaneous water use efficiency (WUE_inst_) at 75% pod formation stage and Agronomic water use efficiency (WUE_y_) at maturity of four guar genotypes under five irrigation regimes from 2016 to 2018, Las Cruces, NM.

	Intrinsic Water Use Efficiency (µmol m^−2^ s^−1^/mol m^−2^ s^−1^)			Instantaneous Water Use Efficiency (µmol m^−2^ s^−1^/mmol m^−2^ s^−1^)			Agronomic Water Use Efficiency (kg ha^−1^/mm^−1^)		
	75% Pod Formation Stage			75% Pod Formation Stage			Maturity		
	2016	2017	2018	2016	2017	2018	2016	2017	2018
Irrigation regime (I)									
I_1_	45.4	44.9	58.6	4.2	2.9 bc	2.7	10.9 c	5.6	2.9 ab
I_2_	47.6	34.9	66.2	4.4	2.8 c	2.6	11.0 c	5.3	2.2 c
I_3_	47.3	38.6	93.6	4.5	2.8 c	2.9	12.4 bc	5.3	2.3 bc
I_4_	53.4	46.1	87.4	4.5	3.0 ab	2.8	13.5 b	5.3	2.3 bc
I_5_	44.7	47.7	72.8	4.3	3.2 a	2.6	18.1 a	5.9	2.9 a
Standard Error	3.3	5.7	10.3	0.4	0.2	0.1	0.6	0.3	0.2
Genotype (G)									
Kinman	50.9	40.9	74.1	4.5	2.7	2.7	13.6 a	5.7	2.7
Lewis	47.2	38.2	73.8	4.5	3.1	2.7	13.3 a	5.0	2.4
Matador	47.4	45.4	76.0	4.4	3.0	2.8	13.6 a	5.6	2.7
NMSU-15-G1	45.2	45.2	79.0	4.2	3.0	2.7	12.3 b	5.4	2.2
Standard Error	3.1	5.0	7.4	0.4	0.2	0.1	0.5	0.3	0.2
Interaction (I × G)	NS	NS	NS	NS	NS	NS	NS	NS	NS

Means within a column and particular effects not including letters or sharing a letter do not differ at α < 0.05 (Fisher’s F-protected LSD). I_1_-normal irrigation, I_2_-no irrigation at 75% pod formation, I_3_-no irrigation at 50% and 75% pod formation, I_4_-terminate irrigation at flowering, and I_5_-terminate irrigation at flowering + biogenic silica. The 50%, 75%, and 100% pod formation stages at 57, 72, and 100 days after planting, respectively; maturity stage at 120 days after planting. NS = Not significant at α < 0.05.

**Table 4:** Number of clusters per plant, pods per plant, seeds per pod, and seeds per plant of four guar genotypes under five irrigation regimes from 2016 to 2018, Las Cruces, NM.

	Clusters per Plant			Pods per Plant			Seeds per Pod			Seeds per Plant		
	2016	2017	2018	2016	2017	2018	2016	2017	2018	2016	2017	2018
Irrigation regime (I)												
I_1_	66.5 b	47.6	30.9	279.6 ab	226.7 a	119.2 a	7.1	8.5	7.6	2006 ab	1918 a	894
I_2_	52.8 b	40.5	23.2	208.9 b	195.6 abc	76.4 b	7.1	8.2	7.1	1490 b	1602 ab	554
I_3_	58.4 b	36.7	22.0	231.5 b	158.2 c	66.6 b	7.1	8.5	6.7	1639 b	1346 b	461
I_4_	64.9 b	41.9	17.8	258.5 b	178.7 bc	60.5 b	7.2	8.4	7.0	1848 b	1508 b	432
I_5_	84.5 a	48.1	27.2	350.8 a	223.1 ab	95.8 ab	7.3	8.6	7.0	2593 a	1938 a	661
Standard Error	5.4	4.4	4.8	27.9	18.2	18.8	0.1	0.1	0.2	206	137	153
Genotype (G)												
Kinman	74.2 a	50.3	25.5	266.1	195.4	90.0	6.7 c	8.1 b	6.9 b	1778	1585	639
Lewis	63.0 ab	43.6	24.2	270.5	215.8	86.3	7.4 a	8.4 a	6.9 b	2005	1833	589
Matador	53.9 b	40.0	23.1	246.1	209.4	84.8	7.6 a	8.6 a	7.3 a	1870	1794	631
NMSU-15-G1	70.5 a	37.9	24.1	280.7	165.4	73.7	7.1 b	8.6 a	7.2 ab	2009	1437	543
Standard Error	4.2	4.3	4.4	22.8	17.3	15.9	0.1	0.1	0.2	170	133	125
Interaction (I × G)	NS	NS	NS	NS	NS	NS	NS	NS	NS	NS	NS	NS

Means within a column and particular effects not including letters or sharing a letter do not differ at α < 0.05 (Fisher’s F-protected LSD). I_1_-normal irrigation, I_2_-no irrigation at 75% pod formation, I_3_-no irrigation at 50% and 75% pod formation, I_4_-terminate irrigation at flowering, and I_5_-terminate irrigation at flowering + biogenic silica. The 50% and 75% pod formation stages at 57 and 72 days after planting, respectively. NS = Not significant at α < 0.05.

**Table 5:** One thousand-seed weight, harvest index, and seed yield of four guar genotypes under five irrigation regimes from 2016 to 2018, Las Cruces, NM.

	1000-Seed Weight (g)			Harvest Index			Seed Yield (kg ha^−1^)		
	2016	2017	2018	2016	2017	2018	2016	2017	2018
Irrigation regime (I)									
I_1_	36.3	34.6 ab	36.7	0.43 a	0.29 bc	0.25	2696 a	2908	2542 a
I_2_	36.4	32.3 c	35.5	0.44 a	0.28 c	0.22	2314 b	2622	1512 b
I_3_	35.7	33.3 c	35.3	0.44 a	0.31 ab	0.21	2253 b	2543	1447 b
I_4_	35.9	33.5 bc	35.1	0.44 a	0.32 a	0.22	2163 b	2436	1399 b
I_5_	35.2	35.1 a	35.1	0.40 b	0.32 a	0.25	2913 a	2717	1778 b
Standard Error	0.5	0.4	0.6	0.01	0.01	0.02	107	157	145
Genotype (G)									
Kinman	35.1 bc	32.7 b	36.1 a	0.43	0.33 a	0.27 a	2543 a	2763	1845
Lewis	36.1 b	33.5 b	35.6 a	0.43	0.29 b	0.23 b	2476 a	2453	1668
Matador	34.8 c	33.1 b	33.7 b	0.43	0.30 ab	0.23 b	2560 a	2736	1876
NMSU-15-G1	37.5 a	35.8 a	36.7 a	0.43	0.29 b	0.19 c	2291 b	2629	1554
Standard Error	0.4	0.5	0.6	0.01	0.01	0.01	86	140	130
Interaction (I × G)	NS	NS	NS	NS	NS	NS	NS	NS	NS

Means within a column and particular effects not including letters or sharing a letter do not differ at α < 0.05 (Fisher’s F-protected LSD). I_1_-normal irrigation, I_2_-no irrigation at 75% pod formation, I_3_-no irrigation at 50% and 75% pod formation, I_4_-terminate irrigation at flowering, and I_5_-terminate irrigation at flowering + biogenic silica. The 50% and 75% pod formation stages at 57 and 72 days after planting, respectively. NS = Not significant at α < 0.05.

## Data Availability

All data supporting this study are included in the article.
